# Synergetic enhancement of gold nanoparticles and 2-mercaptobenzothiazole as highly-sensitive sensing strategy for tetrabromobisphenol A

**DOI:** 10.1038/srep26044

**Published:** 2016-05-17

**Authors:** Xuerong Chen, Liudi Ji, Yikai Zhou, Kangbing Wu

**Affiliations:** 1 Key Laboratory of Environment and Health, Ministry of Education, School of Public Health, Tongji Medical College, Huazhong University of Science and Technology, Wuhan 430030, China; 2 Key Laboratory for Material Chemistry of Energy Conversion and Storage, Ministry of Education, School of Chemistry and Chemical Engineering, Huazhong University of Science and Technology, Wuhan 430074, China

## Abstract

Various gold nanoparticles (AuNPs) were *in-situ* prepared on the electrode surface through electrochemical reduction under different potentials such as −0.60, −0.50, −0.40, −0.30 and −0.20 V. The reduction potentials heavily affect the surface morphology and electrochemical activity of AuNPs such as effective area and catalytic ability, as confirmed using atomic force microscopy and electrochemical impedance spectroscopy. The electrochemical behaviors of tetrabromobisphenol A (TBBPA), a widely-existed pollutant with severe adverse health effects, were studied. The oxidation activity of TBBPA enhances obviously on the surface of AuNPs, and the signal improvements of TBBPA show difference on the prepared AuNPs. Interestingly, the existence of 2-mercaptobenzothiazole (MBT) further improves the oxidation signals of TBBPA on AuNPs. The synergetic enhancement effects of AuNPs and MBT were studied using cyclic voltammetry and chronocoulometry. The numerous nano-scaled gold particles together with the strong hydrophobic interaction between TBBPA and the assembled MBT on AuNPs jointly provide highly-effective accumulation for TBBPA. As a result, a sensitive and simple electrochemical method was developed for the direct determination of TBBPA, with detection limit of 0.12 μg L^−1^ (0.22 nM). The practical applications in water samples manifest that this new sensing system is accurate and feasible.

Due to wide applications in textiles, printed circuit boards and electronic equipments as brominated flame retardant (BFR), tetrabromobisphenol A (TBBPA) has been detected not only in the environmental samples^1^,^2^, but also in wildlife^3^ and humans^4^,^5^. Studies have proved that overexposure to TBBPA can cause severe adverse health effects. For example, TBBPA has been identified as an endocrine disruptor because it shows 10-fold increase in binding affinity to human transthyretin compared with the natural thyroid hormone thyroxin^6^. In addition, the immunotoxicity^7^, nephrotoxicity^8^ and developmental toxicity^9^ of TBBPA have also been proven. Therefore, determination of TBBPA in a rapid, sensitive and simple manner is quite important. Electrochemical sensing has obtained more and more attention due to its unique properties such as sensitivity, rapidness, good handling convenience, low cost, *in situ* monitoring and miniaturization. Until now, indirect electrochemical methods have been developed for the determination of TBBPA using [Fe(CN)_6_]^3−/4−^ as the indicator^10^ or based on the response signals of [Fe(CN)_6_]^3−^-TBBPA complex^11^,^12^. So the direct electrochemical determination of TBBPA is urgent and still full of challenge.

Owing to their fascinating electrical, chemical, optical and catalytic properties, gold nanostructures have obtained wide applications in electrochemical sensing^13^,^14^,^15^,^16^, catalysis^17^,^18^, surface-enhanced Raman scattering^19^,^20^ and so on. Until now, various methods have been developed for the preparation of Au nanostructures, including chemical reduction^21^,^22^, seed-growth^23^, vapor deposition^24^, photochemical reduction^25^, hydrothermal synthesis^26^ and electrochemical deposition^27^. Among them, electrochemical preparation is considered as a simple and efficient strategy to precisely tune the shape of Au nanostructures.

Herein, a series of gold nanoparticles (AuNPs) were prepared on the surface of glassy carbon electrode (GCE) *via* one-step electroreduction at different potentials such as −0.60, −0.50, −0.40, −0.30 and −0.20 V. It is found that the reduction potentials have big impacts on the surface morphology, effective sensing area and catalytic ability of AuNPs. Moreover, we also find that the oxidation activity of TBBPA on the surface of different AuNPs is also controlled by the deposition potentials. The AuNPs that prepared at −0.40 V are more sensitive for the oxidation of TBBPA, and exhibits stronger enhancement effects. Interestingly, we notice that the oxidation signals of TBBPA further increase greatly on the surface of AuNPs after addition of 2-mercaptobenzothiazole (MBT) in the buffer solution. The synergetic enhancement mechanism of AuNPs and MBT has been discussed, and a novel electrochemical sensing platform with high sensitivity has been developed for the direct determination of TBBPA. The developed sensing system is promising in the automatic and on-line monitoring TBBPA because the preparation of sensing film is fulfilled by electrochemical processes.

## Results and Discussion

### Properties of Different AuNPs

**Figure 1:**
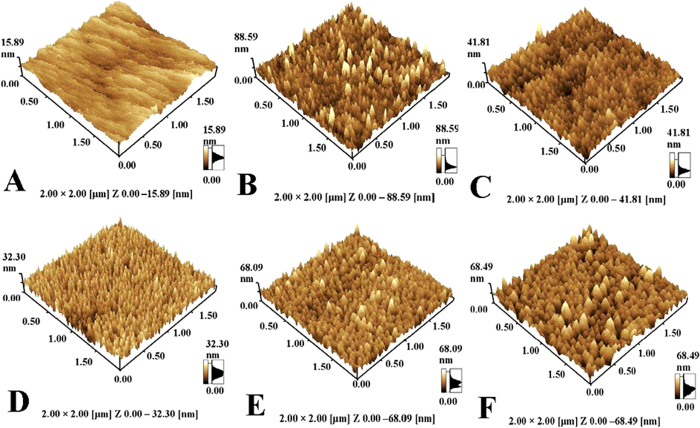
AFM images of GCE (**A**) and AuNPs modified GCEs that prepared at −0.60 V (**B**), −0.50 V(**C**), −0.40 V (**D**), −0.30 V (**E**) and −0.20 V (**F**).

AFM was used to characterize the detailed three-dimensional structures of different AuNPs. The unmodified GCE surface is very smooth (Fig. 1A), and a large number of nanoparticles are observed on GCE surface after electrochemical reduction of HAuCl_4_ (Fig. 1B–F). The coating of AuNPs not only enhances the surface roughness of GCE, but also forms abundant three-dimensional structures. In addition, we clearly find that the particle size and surface arrangements of AuNPs are heavily dependent on the reduction potentials. From the comparison, it is apparent that the surface morphology of AuNPs that deposited at −0.40 V is more attractive due to smaller particle size and more uniform arrangements. In conclusion, the different surface morphology manifests that the shape of AuNPs can be easily controlled by variation of the reduction potentials.

**Figure 2:**
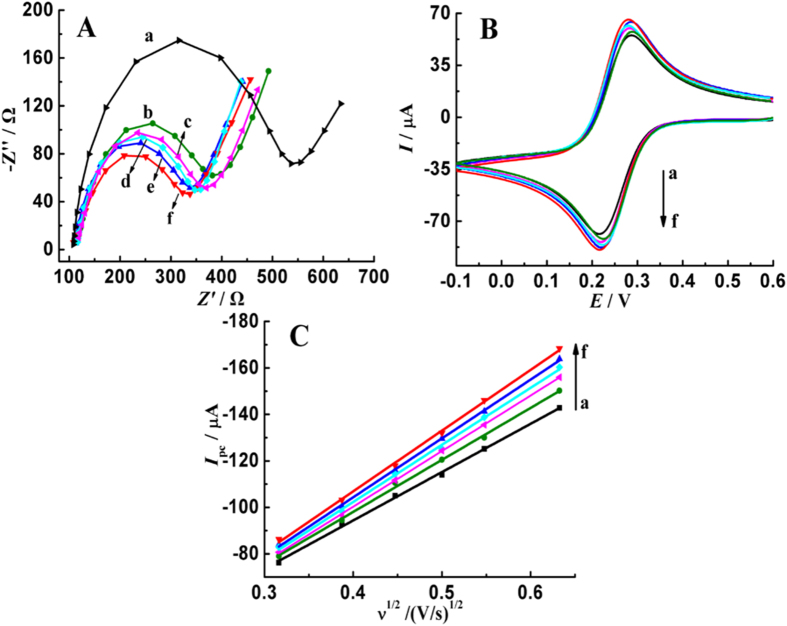
(**A**) Nyquist impedance plots of 5.0 mM K_3_/K_4_Fe(CN)_6_ in 0.1 M KCl on GCE (a) and AuNPs modified GCEs that prepared at −0.60 (b), −0.20 (c), −0.30 (d), −0.50 (e) and −0.40 V (f), frequency range: 100 kHz to 1 Hz, amplitude: 5 mV; (**B**) Cyclic voltammograms of 5.0 mM K_3_[Fe(CN)_6_] in 1.0 M KCl on GCE (a) and AuNPs modified GCEs that prepared at −0.60 (b), −0.20 (c), −0.30 (d), −0.50 (e) and −0.40 V (f), scan rate, 100 mV s^−1^; (**C**) Variation of reduction peak currents of 5.0 mM K_3_[Fe(CN)_6_] with the square root of scan rate on on GCE (a) and AuNPs modified GCEs that prepared at −0.60 (b), −0.20 (c), −0.30 (d), −0.50 (e) and −0.40 V (f).

The electron exchange ability of different AuNPs was compared using electrochemical impedance spectroscopy (EIS). As depicted in Fig. 2A, well-shaped semicircles with different diameter are clearly observed over the high frequency range, and straight lines appear in the low frequency range. Compared with those on bare GCE, the diameter of semicircles decreases obviously on the surface of AuNPs modified GCEs. Based on the Randles equivalent circuit, the determination of the values of charge transfer resistance (*R*_ct_) is repeated 3 times, and the results are fitted to be 311.2 ± 15.3, 261.3 ± 11.9, 169.8 ± 6.5, 152.9 ± 8.2, 178.4 ± 7.3 and 222.4 ± 9.1 Ω on bare GCE and AuNPs modified GCEs that prepared at −0.60, −0.50, −0.40, −0.30 and −0.20 V. The decreased *R*_ct_ value reveals that AuNPs exhibit higher catalytic ability, and the AuNPs that prepared at −0.40 V are superior.

In addition, the effective response area of different GCEs were measured using the probe of K_3_[Fe(CN)_6_]. As shown in Fig. 2B, a pair of well-defined redox peaks with peak potential separation (Δ*E*_p_) of 69 mV is observed, and the peak currents increase on the surface of AuNPs modified GCEs. When increasing the scan rate (*v*) from 0.1 to 0.4 V s^−1^, the peak potentials remain unchanged, but the reduction peak currents (*I*_pc_) increase linearly with the square root of scan rate, as displayed in Fig. 2C, suggesting a reversible and diffusion-controlled electrode process. According to the Randles-Sevcik equation, the effective electrode areas (*A*) are individually calculated to be 0.0564, 0.0625, 0.0682, 0.0704, 0.0665 and 0.0653 cm^2^ for bare GCE and AuNPs modified GCEs that prepared at −0.60, −0.50, −0.40, −0.30 and −0.20 V. Apparently, the deposition of AuNPs on GCE surface enhances the electrochemical sensing area, and the values of AuNPs that prepared at −0.40 V are larger. In summary, the used reduction potentials not only influence the surface morphology of AuNPs, but also affect the electron transfer ability and electrochemical sensing area of AuNPs.

### Synergetic Enhancement Effects of AuNPs and MBT

**Figure 3:**
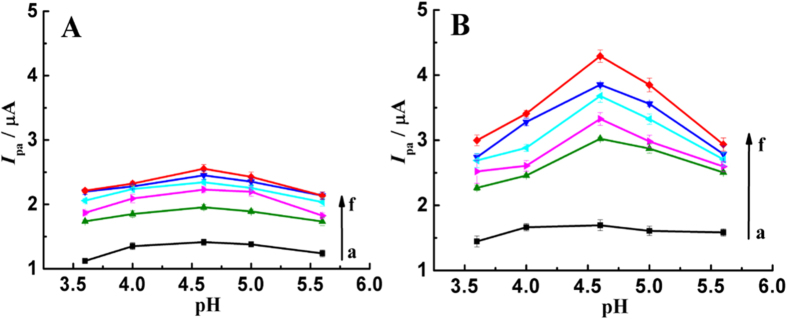
Oxidation peak currents of 0.5 mg L^−1^ TBBPA at different pH values on GCE (a) and AuNPs modified GCEs that prepared at −0.60 (b), −0.20 (c), −0.30 (d), −0.50 (e) and −0.40 V (f) in the absence (**A**) and presence of 2.0 μM MBT (**B**), scan rate: 100 mV s^−1^.

The electrochemical behaviors of TBBPA in 0.1 M acetate buffer solutions with different pH values were individually examined using cyclic voltammetry (CV). On the surface of bare GCE and AuNPs modified GCEs that prepared at −0.60, −0.50, −0.40, −0.30 and −0.20 V, just an oxidation wave is observed for TBBPA, and the peak currents enhance obviously on the surface of AuNPs modified GCEs, as Fig. 3A shown. The signal enlargements manifest that the prepared AuNPs are more active for the oxidation of TBBPA, and moreover, the deposition potentials for AuNPs have big impacts as confirmed from the different oxidation peak currents of TBBPA. In addition, we clearly find that the oxidation activity of TBBPA on GCE and AuNPs is pH-dependent because the oxidation signals increase gradually with pH value from 3.6 to 4.6, and then gradually decrease with further improving pH value to 5.6. Similarly, it is also found that the enhancement activity of AuNPs toward TBBPA oxidation is also related to pH value, and the enhancement effects are much higher at pH of 4.6.

Figure 3B shows the oxidation peak currents of TBBPA on the surface of bare GCE and AuNPs modified GCEs in different acetate buffer solutions containing 2.0 μM MBT. The oxidation peak currents of TBBPA are also pH-dependent, and much higher at pH of 4.6. Due to larger surface area and higher electron transfer ability, the prepared AuNPs exhibit notable signal enhancement ability toward TBBPA oxidation, and consequently enhance its oxidation peak currents obviously. Additionally, the oxidation activity of TBBPA in the presence of MBT is also pH-dependent, and the signal is much higher at pH 4.6. However, it is must to be indicated that the oxidation signals of TBBPA on the AuNPs modified GCEs further enhance obviously in the presence of MBT, compared with the values in Fig. 3A. *Via* formation of Au-S bond, the MBT molecules can be firmly assembled on the surface of AuNPs, and the benzothiazole ring then display strong hydrophobic interaction with TBBPA. As a result, AuNPs and MBT exhibit synergetic enhancement effects for the oxidation of TBBPA, and greatly enhance the response signals of TBBPA. Additionally, we also find that the synergetic enhancement ability of AuNPs and MBT is also controlled by pH value, and is much stronger at pH of 4.6.

**Figure 4:**
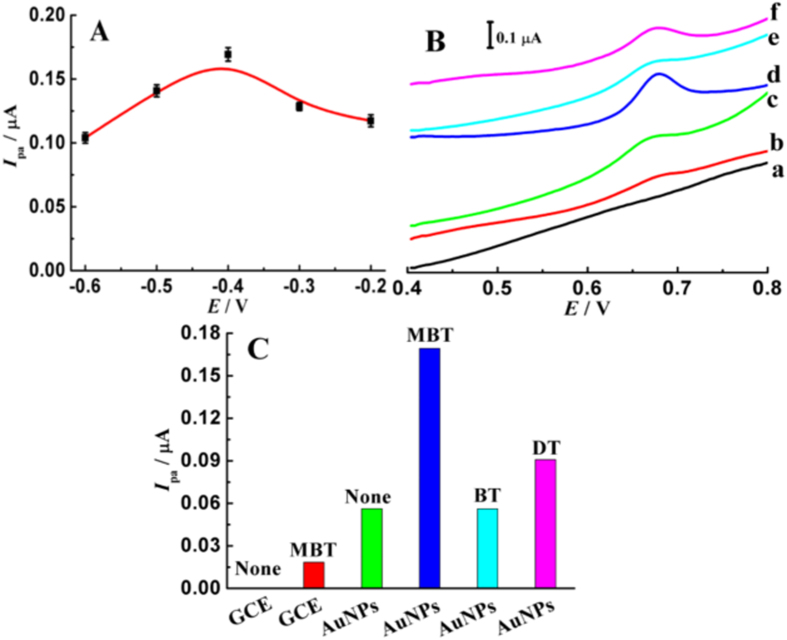
(**A**) Influences of reduction potential of AuNPs on the oxidation peak currents of 5.0 μg L^−1^ TBBPA in the presence of 2.0 μM MBT; (**B**) DPV curves of 5.0 μg L^−1^ TBBPA on GCE (a, b), AuNPs modified GCE (c–f) in pH 4.6 acetate buffer (a, c) and in the presence of 2.0 μM MBT (b, d), BT (e) and DT (f); accumulation time: 3 min.

To further discuss the enhancement effects of AuNPs and MBT toward trace levels of TBBPA, the oxidation behaviors of 5.0 μg L^−1^ TBBPA were examined using differential pulse voltammetry (DPV). From the property analysis, we know that the surface morphology and electrochemical activity of AuNPs are controlled by the reduction potentials. So we speculate that the reduction potential also affects the signal enhancement ability of AuNPs toward TBBPA oxidation. This speculation is proved using Fig. 4A, and it is found that the obtained AuNPs using different potentials exhibit different enhancement effects toward the oxidation signals of TBBPA. From Fig. 4A, we clearly find that the prepared AuNPs at −0.40 V is more active for TBBPA oxidation, most likely resulted from higher electron transfer ability and larger electrochemical sensing area. Therefore, the AuNPs that prepared at −0.40 V were used to construct sensing platform for TBBPA.

Figure 4B,C illustrate the synergetic enhancement effects of AuNPs and MBT toward TBBPA oxidation. On the surface of bare GCE, no oxidation wave is observed for 5.0 μg L^−1^ TBBPA even after 3-min accumulation in pH 4.6 acetate buffer (curve a), and a very low oxidation peak appears at 0.676 V after addition of 2.0 μM MBT (curve b). However, the oxidation wave of TBBPA is improved obviously on the surface of AuNPs modified GCE that prepared at −0.40 V (curve c), and further improved greatly in the presence of 2.0 μM MBT (curve d). Additionally, the oxidation behaviors of TBBPA in the presence of benzothiazo (BT) and 1-dodecanethiol (DT) were also investigated to deeply elucidate the synergetic enhancement effects of AuNPs and MBT. As seen from the curve (e) in Fig. 4B, we clearly find that the oxidation signals of TBBPA on AuNPs that prepared at −0.40 V just increase slightly in the presence of BT, revealing that the enhancement ability of BT is very weak. When adding low concentration of DT that contains -SH and long C-H chain, the oxidation wave of TBBPA enhance obviously on the surface of AuNPs, as shown in the curve (f) in Fig. 4B. Without -SH group, the BT molecules can not adsorb on the surface of AuNPs, while the DT molecules can be effectively assembled on AuNPs through Au-S bond. Therefore, DT exhibits effective enhancement ability toward TBBPA oxidation, while the enhancement effects of BT are very poor. Moreover, we also find that MBT has stronger enhancement activity toward TBBPA compared with DT, maybe attributed to the stronger hydrophobic interactions resulted from benzothiazole ring and TBBPA. In conclusion, the great signal improvements manifest that AuNPs and MBT possess remarkable synergetic enhancement ability for TBBPA oxidation, and the detection sensitivity is certainly enhanced greatly.

### Enhancement Mechanism of AuNPs and MBT

**Figure 5:**
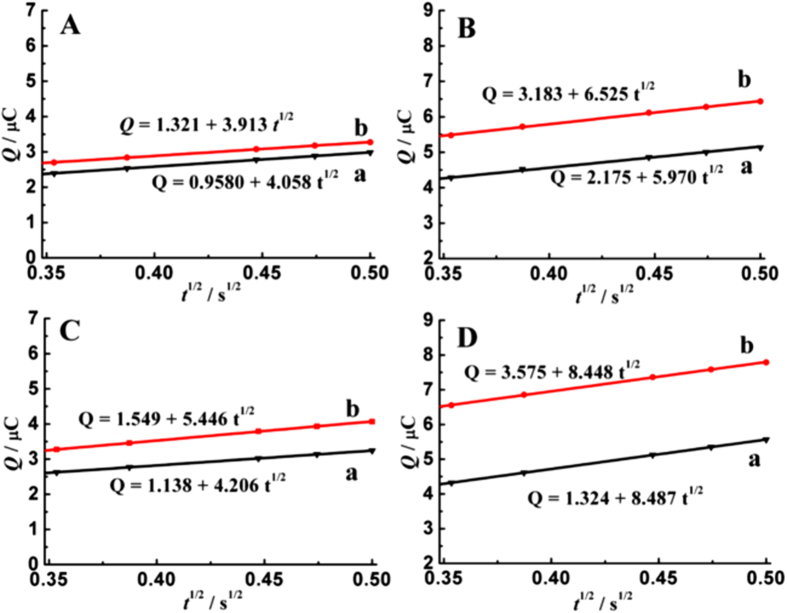
*Q*-t^1/2^ plots on GCE (**A**) and AuNPs modified GCE (**B**) in pH 4.6 acetate buffer (curve a) and containing 0.5 mg L^−1^ TBBPA (curve b); *Q*-t^1/2^ plots on GCE (**C**) and AuNPs modified GCE (**D**) in pH 4.6 acetate buffer containing 2.0 μM MBT (curve a) and in the presence of 0.5 mg L^−1^ TBBPA (curve b).

To elucidate the origin of enhancement effects for TBBPA oxidation, the adsorption behaviors of TBBPA on different GCEs were studied using chronocoulometry. During the potential step from 0.40 to 0.80 V, the curves of charge (*Q*)-time (*t*) were firstly recorded, and then transferred to *Q*-*t*^1/2^ straight lines. Figure 5 shows the *Q*-*t*^1/2^ plots on GCE (A) and AuNPs modified GCE (B) in pH 4.6 acetate buffer (curve a) and in the presence of TBBPA (curve b). After addition of MBT, the *Q*-*t*^1/2^ plots on GCE (Fig. 5C) and AuNPs modified GCE (Fig. 5D) were also recorded in pH 4.6 acetate buffer (curve a) and in the presence of TBBPA (curve b). According to the integrated Cottrell equation, the intercept value of *Q*-*t*^1/2^ plot in pH 4.6 acetate buffer represents the charge of double-layer capacitance (*Q*_dl_), while the intercept value in the presence of TBBPA is the summation of *Q*_dl_ and *Q*_ads_ (the Faradaic charge of adsorbed species)^28^. So the *Q*_ads_ values of TBBPA on GCE in the absence of MBT are calculated to be 0.363 μC, and then increase to 0.411 μC in the presence of MBT, as individually confirmed from Fig. 5A,C. On the surface of AuNPs modified GCE that prepared at −0.40 V, the *Q*_ads_ values in the absence of and presence of MBT are to be 1.008 and 2.251 μC, as obtained from Fig. 5B,D. Clearly, the surface modification of AuNPs greatly improves the accumulation ability of TBBPA, and of course, results in higher response signals for TBBPA oxidation. On the other hand, the existence of MBT also enhances the surface accumulation efficiency of TBBPA on GCEs, especially on the AuNPs modified GCE. The numerous nano-scaled gold particles on GCE surface together with the strong hydrophobic interaction between TBBPA and MBT that assembled on AuNPs jointly provide highly-effective accumulation for TBBPA, and then greatly improves the surface amount of TBBPA. As a result, the oxidation signals and detection sensitivity of TBBPA enhance significantly.

### Electrochemical Determination of TBBPA

**Figure 6:**
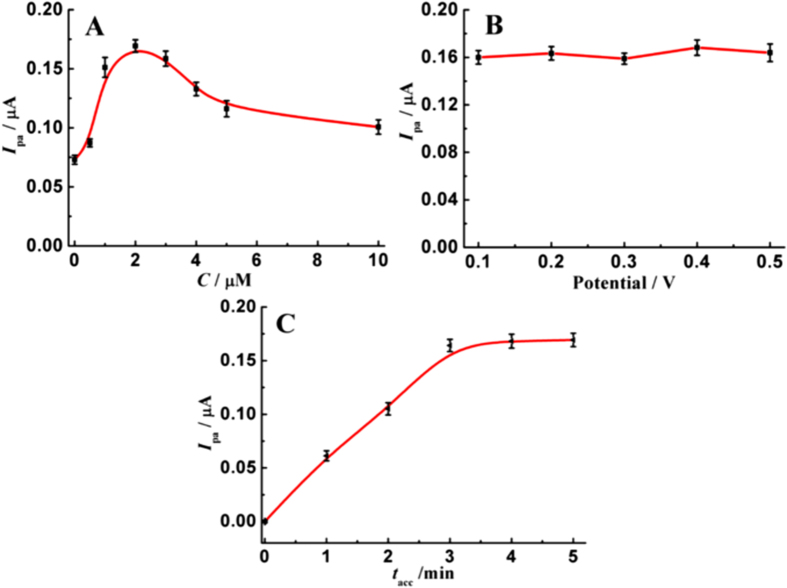
Influences of MBT concentration (**A**), accumulation potential (**B**), and accumulation time (**C**) on the oxidation peak currents of 5.0 μg L^−1^ TBBPA on AuNPs modified GCE that prepared at −0.40 V.

Figure 6A demonstrates the effects of concentration of MBT on the oxidation signals of TBBPA. As increasing the MBT concentration from 0 to 2.0 μM, the oxidation peak currents of TBBPA on AuNPs modified GCE increase greatly, attributed to the obviously-improved accumulation efficiency resulted from the hydrophobic interaction between TBBPA and MBT. With further improving MBT concentration to 10.0 μM, the oxidation peak currents of TBBPA decrease gradually, maybe ascribed to the fact that too much MBT lowers the electric conductivity. Therefore, the concentration of added MBT is fixed at 2.0 μM.

The variation of oxidation peak currents of TBBPA with the accumulation potentials was measured, as shown in Fig. 6B. It is found that the influence of accumulation potential is slight because the oxidation signals of TBBPA vary slightly. For handling convenience and better wave shape, the accumulation potential and initial potential were controlled at 0.40 V. However, accumulation time has big impacts. As seen in Fig. 6C, the oxidation peak currents of TBBPA increase rapidly with improving accumulation time from 0 to 3.0 min, and then enhance slightly. Considering response signals and analysis time, 3-min accumulation was used before the DPV scans from 0.40 V to 0.80 V.

The reproducibility between multiple AuNPs modified GCEs was evaluated using 5.0 μg L^−1^ TBBPA. The value of relative standard deviation (RSD) is 4.2% for nine GCEs, revealing good fabrication reproducibility and detection precision.

The potential interferences of other phenols on the detection of TBBPA were investigated under the optimized conditions, as shown in Fig. S1 (Supporting Information). The phenols with close oxidation peak potential (*E*_pa_) such as bisphenol A (BPA, *E*_pa_ = 0.676 V), p-cresol (*E*_pa_ = 0.688 V), o-chlorophenol (*E*_pa_ = 0.744 V), p-chlorophenol (*E*_pa_ = 0.768 V) and phenol (*E*_pa_ = 0.800 V) have no influences on the oxidation signals of 5.0 μg L^−1^ TBBPA (9.2 nM, *E*_pa_ = 0.672 V) because their oxidation signals are much lower, even in the concentration of 100 nM. In addition, the phenols with much lower or higher oxidation peak potentials such as hydroquinone (*E*_pa_ = 0.352 V), catechol (*E*_pa_ = 0.392 V), p-nitrophenol (*E*_pa_ = 1.212 V) and o-nitrophenol (*E*_pa_ = 1.260 V) also do not interfere with the signals of TBBPA because their oxidation signals at 100 nM are very low. The good selectivity for TBBPA may be attributed to much stronger interaction between TBBPA and MBT that resulted from hydrophobic and conjugate force.

**Figure 7:**
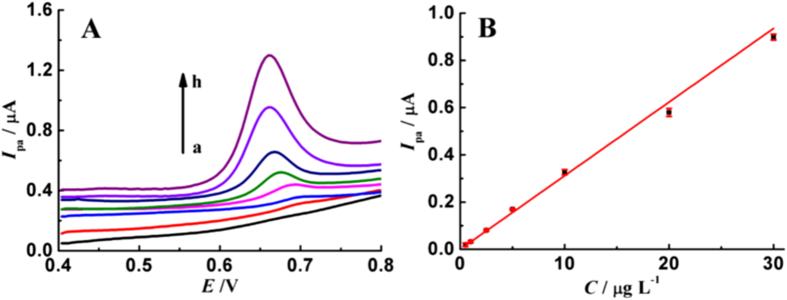
(**A**) DPV curves of TBBPA on AuNPs modified GCE in the presence of 2.0 μM MBT with concentrations of 0 (a), 0.5 (b), 1 (c), 2.5 (d), 5 (e), 10 (f), 20 (g), 30 μg L^−1^ (h); (**B**) Calibration curve for TBBPA. Error bar represents the standard deviation of triple measurements.

Figure 7 illustrates the linear range for the determination of TBBPA using AuNPs modified GCE in presence of MBT. The oxidation peak currents (*I*_pa_, μA) increase linearly with the concentration of TBBPA (*C*, μg L^−1^) over the range from 0.5 to 30 μg L^−1^, and the linear regression equation is expressed as *I*_pa_ = 0.03115 *C.* The correlation coefficient (*R*) is 0.998, revealing good linearity. After 3-min accumulation, the value of detection limit is estimated to be 0.12 μg L^−1^ (0.22 nM) based on three signal-to-noise ratio.

### Practical Application

In order to evaluate the practical applications, the proposed method was used to determine TBBPA in different water samples that collected in Wuhan city. The samples were filtered using 0.45 μm cellulose acetate membrane before analysis. After addition of 5.0 mL sample solution into 5.0 mL pH 4.6 acetate buffer containing 4.0 μM MBT, the DPV curves were recorded from 0.40 to 0.80 V after 3-min accumulation. Each sample solution undergoes three parallel detections, and the RSD is below 3.7%, revealing good precision. The content was obtained by the standard addition method, and the results were listed in Table 1. In order to testify the accuracy, TBBPA standards were spiked into the original water samples, and then analyzed according to above procedure. The values of recovery are in the ranger from 97.2~103.6%, revealing that the developed determination method for TBBPA is accurate and has promising application.

## Conclusions

By means of changing reduction potentials, gold nanoparticles with different morphology and electrochemical activity have been prepared. The oxidation activity of TBBPA enhances effectively on the surface of AuNPs, and further increases greatly in the presence of MBT. The abundant nano-scaled gold particles together with the strong hydrophobic interaction resulted from TBBPA and the assembled MBT on AuNPs jointly improve the accumulation efficiency of TBBPA. Moreover, the prepared AuNPs using different potentials exhibit different electrochemical reactivity toward TBBPA oxidation, and the AuNPs that deposited at −0.40 V is superior. Utilizing the strong synergetic enhancement effects of AuNPs and MBT, a highly-sensitive, accurate and simple sensing platform has been successfully developed for the direct electrochemical determination of TBBPA.

## Methods

### Reagents

All chemicals were of analytical grade and used as received. 2.0 mg mL^−1^ stock solution of TBBPA (Laboratories of Dr. Ehrenstorfer, German) was prepared with ethanol, and stored at 4 °C. HAuCl_4_ was purchased from Sinopharm Chemical Reagent Co., Ltd. (Shanghai, China). MBT, BT and DT were purchased from Sigma-Aldrich, and individually dissolved into ethanol in concentration of 2.0 mM. Ultrapure water (18.2 MΩ) was obtained from a Milli-Q water purification system and used throughout.

### Instruments

Electrochemical measurements were performed on a CHI 660A electrochemical workstation (Chenhua Instrument, Shanghai, China) with a conventional three-electrode system. The working electrode is a modified glassy carbon electrode (GCE), the reference electrode is a saturated calomel electrode (SCE), and the counter electrode is a platinum wire. Atomic force microscopy (AFM) was performed with a SPM 9700 microscope (Shimadzu, Japan).

### Electrochemical Preparation of AuNPs

Prior to electrodeposition, the GCE was polished with 0.05 μm alumina slurry, and ultrasonically washed in ultrapure water. After that, AuNPs were deposition on the surface of clear GCE under different potentials for 200 s in 0.5 M H_2_SO_4_ containing 0.5 mM HAuCl_4_. The used reduction potentials were −0.60 V, −0.50 V, −0.40 V, −0.30 V and −0.20 V. Finally, the resulting electrode was rinsed with ultrapure water to remove any adsorbed species.

### Analytical Procedure for TBBPA

0.1 M, pH 4.6 acetate buffer solution containing 2.0 μM MBT was used as supporting electrolyte for TBBPA detection. The differential pulse voltammograms (DPV) were recorded from 0.40 to 0.80 V after 3-min accumulation, and the oxidation peak currents at 0.672 V were measured for TBBPA. The pulse amplitude was 50 mV, the pulse width was 50 ms, and the scan rate was 40 mV s^−1^.

## Additional Information

**How to cite this article**: Chen, X. *et al.* Synergetic enhancement of gold nanoparticles and 2-mercaptobenzothiazole as highly-sensitive sensing strategy for tetrabromobisphenol A. *Sci. Rep.* **6**, 26044; doi: 10.1038/srep26044 (2016).

## Supplementary Material

Supplementary Information

## Figures and Tables

**Table 1:** Detection of TBBPA in water samples.

No.	Measured (μg L^−1^)	Added (μg L^−1^)	Found (μg L^−1^)	Recovery
1	1.03	1.00	2.05	102.0%
2	1.93	2.00	3.91	99.0%
3	4.80	5.00	9.98	103.6%
4	7.55	7.50	14.84	97.2%
5	10.05	10.00	19.81	97.6%
