# Supplementary material for: Synergetic enhancement of gold nanoparticles and 2-mercaptobenzothiazole as highly-sensitive sensing strategy for tetrabromobisphenol A
Source: Sci Rep. 2016 May 17;6:26044. doi: 10.1038/srep26044 (PMC4868994; doi:10.1038/srep26044)
Supplement: Supplementary Information [file srep26044-s1.pdf]

SUPPLEMENTARY INFORMATION

Synergetic enhancement of gold nanoparticles and 2-mercaptobenzothiazole as highly-sensitive sensing strategy for tetrabromobisphenol A

Xuerong Chen [1,2], Liudi Ji [2], Yikai Zhou [1*], Kangbing Wu [2*]

[1] Key Laboratory of Environment and Health, Ministry of Education, School of Public Health, Tongji Medical College, Huazhong University of Science and Technology, Wuhan 430030, China

[2] Key Laboratory for Material Chemistry of Energy Conversion and Storage, Ministry of Education, School of Chemistry and Chemical Engineering, Huazhong University of Science and Technology, Wuhan 430074, China

* Corresponding author. K.B. Wu (kbwu@hust.edu.cn)

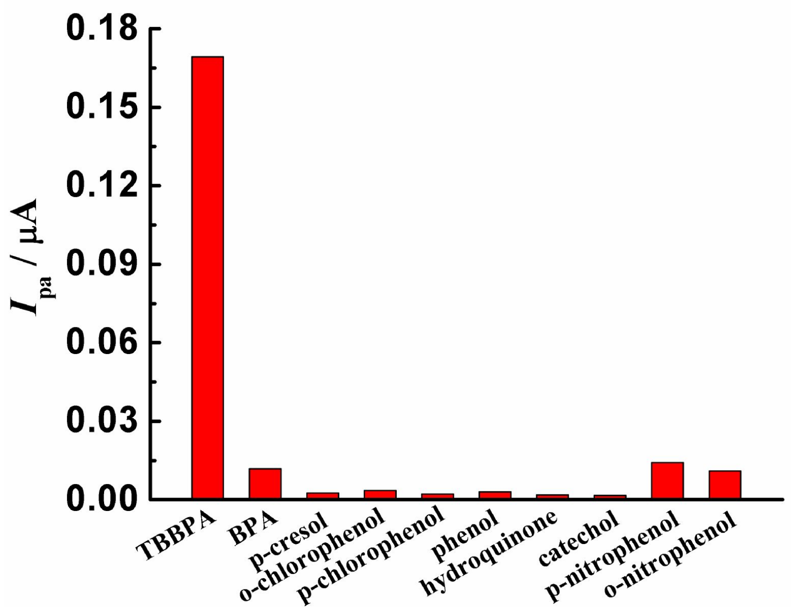

Figure S1. Oxidation peak currents of 9.2 nM TBBPA, 100 nM BPA, p-cresol, o-chlorophenol, p-chlorophenol, phenol, hydroquinone, catechol, p-nitrophenol and o-nitrophenol on AuNPs modified GCE in pH 4.6 buffer containing 2.0 μM MBT.
